# Consecutive Hypoalbuminemia Predicts Inferior Outcome in Patients With Diffuse Large B-Cell Lymphoma

**DOI:** 10.3389/fonc.2020.610681

**Published:** 2021-01-27

**Authors:** Xiaolei Wei, Jingxia Zheng, Zewen Zhang, Qiongzhi Liu, Minglang Zhan, Weimin Huang, Junjie Chen, Qi Wei, Yongqiang Wei, Ru Feng

**Affiliations:** ^1^ Department of Hematology, Nanfang Hospital, Southern Medical University, Guangzhou, China; ^2^ Department of Hematology, The Second Affiliated Hospital of Shantou University Medical College, Shantou, China; ^3^ Department of Hematology, Changsha Central Hospital, South China University, Changsha, China

**Keywords:** diffuse large B-cell lymphoma, albumin, survival, change, outcome

## Abstract

The prognostic value of albumin changes between diagnosis and end-of-treatment (EoT) in diffuse large B-cell lymphoma (DLBCL) remains unknown. We retrospectively analyzed 574 *de novo* DLBCL patients treated with R-CHOP from our and two other centers. All patients were divided into a training cohort (n = 278) and validation cohort (n = 296) depending on the source of the patients. Overall survival (OS) and progression-free survival (PFS) were analyzed by the method of Kaplan–Meier and Cox proportional hazard regression model. In the training cohort, 163 (58.6%) patients had low serum albumin at diagnosis, and 80 of them were present with consecutive hypoalbuminemia at EoT. Patients with consecutive hypoalbuminemia showed inferior OS and PFS (*p* = 0.010 and *p* = 0.079, respectively). Similar survival differences were also observed in the independent validation cohort (*p* = 0.006 and *p* = 0.030, respectively). Multivariable analysis revealed that consecutive hypoalbuminemia was an independent prognostic factor OS [relative risk (RR), 2.249; 95% confidence interval (CI), 1.441–3.509, *p* < 0.001] and PFS (RR, 2.001; 95% CI, 1.443–2.773, *p* < 0.001) in all DLBCL patients independent of IPI. In conclusion, consecutive hypoalbuminemia is a simple and effective adverse prognostic factor in patients with DLBCL, which reminds us to pay more attention to patients with low serum albumin at EoT during follow-up.

## Introduction

The introduction of rituximab into the CHOP regimen (cyclophosphamide, doxorubicin, vincristine, and prednisone) has markedly improved the outcome of diffuse large B-cell lymphoma (DLBCL) (1, 2). Along with this, the prognostic significance of international prognostic index (IPI) was impaired and could only distinguish the low-risk with the high-risk group instead of the four risk groups as previously described (3–5). Alternatively, more and more clinical and biological markers were explored to predict the prognosis of DLBCL, including age, extranodal lesions, cell of origin, c-MYC and Bcl-2 co-expression or translocation, and different biochemical indicators (6–14). As the treatment developed, the prognostic values of these biochemical indicators may also change. It is important to find the new and simple prognostic marker to identify these DLBCL patients with different outcomes throughout the entire treatment.

Our and previous other studies showed that albumin at diagnosis could be used to predict outcome in DLBCL (15–17). Jennifer et al. reported that hypoalbuminemia may predict survival at the start of treatment prior to cycle 2, prior to cycle 4, and 4 weeks after during treatment of DLBCL with R-CHOP (18). However, the prognostic value of albumin changes between diagnosis and end-of-treatment (EoT) in DLBCL remains unknown. Therefore, we performed this study to evaluate the prognostic significance of albumin change after R-CHOP treatment in patients with DLBCL.

## Patients and Methods

### Patients

This multicenter, retrospective study was conducted in three centers. Patients with primary central nervous system and mediastinal lymphoma, immunodeficiency-associated tumors, posttransplant lymphoproliferative disorder and transformed non-Hodgkin lymphoma were excluded from the study. All DLBCL patients were treated with R-CHOP regimen, and only those patients with good response after four cycles of treatment would continue to finish the planned six to eight cycles of R-CHOP treatment. Patients with poor response and progressive disease at interim would transform to second line therapy and high dose therapy with autologous stem cell rescue. A total of 574 patients consecutively diagnosed as *de novo* DLBCL treated with six to eight cycles of R-CHOP between 2003 and 2017 were reviewed. The training cohort consisted of 278 newly diagnosed DLBCL patients treated at Nanfang hospital, and the validation cohort included 296 DLBCL patients at the Second Affiliated Hospital of Shantou University Medical College and Changsha Central Hospital.

### Data Collection

Clinical and treatment data were collected prospectively at different centers and reviewed retrospectively in this study. Clinical data included age, gender, height, weight, serum lactate dehydrogenase (LDH), performance status defined by Eastern Cooperative Oncology Group, number of extranodal sites, disease stage according to the Ann Arbor staging system, IPI score, serum albumin at diagnosis and EoT, and complete blood cell count. GCB and non-GCB subtypes were classified according to the algorithm described by Hans et al. (19). Treatment details were also recorded in electronic medical records system. All patients gave written informed consent themselves prior to treatment allowing the use of their medical records for medical research. This study was approved by the Ethics Committee of Southern Medical University affiliated Nanfang Hospital before study initiation.

### Statistical Analysis

Albumin and overall survival were used as test and state variates in the receiver operating characteristic (ROC) curve, and Youden index was used to determine the best cutoff value of albumin for survival analysis. Distributions of clinical characteristics between the different groups were carried out by Mann–Whitney test or Fisher’s exact test. Overall survival (OS) and progression-free survival (PFS) were analyzed by the Kaplan–Meier method and compared by the log-rank test. OS was defined from the date of diagnosis to death from any cause or the last follow-up. PFS was defined from the date of diagnosis to the date of disease progression, relapse or death from any cause. The multivariable analysis was performed by Cox proportional hazard model. All p values were two-sided, and the significance was defined as p <0.05. Data were analyzed by the Statistical Package of Social Sciences version 13.0 for Windows.

## Results

### Patients’ Characteristics

**Figure 1:**
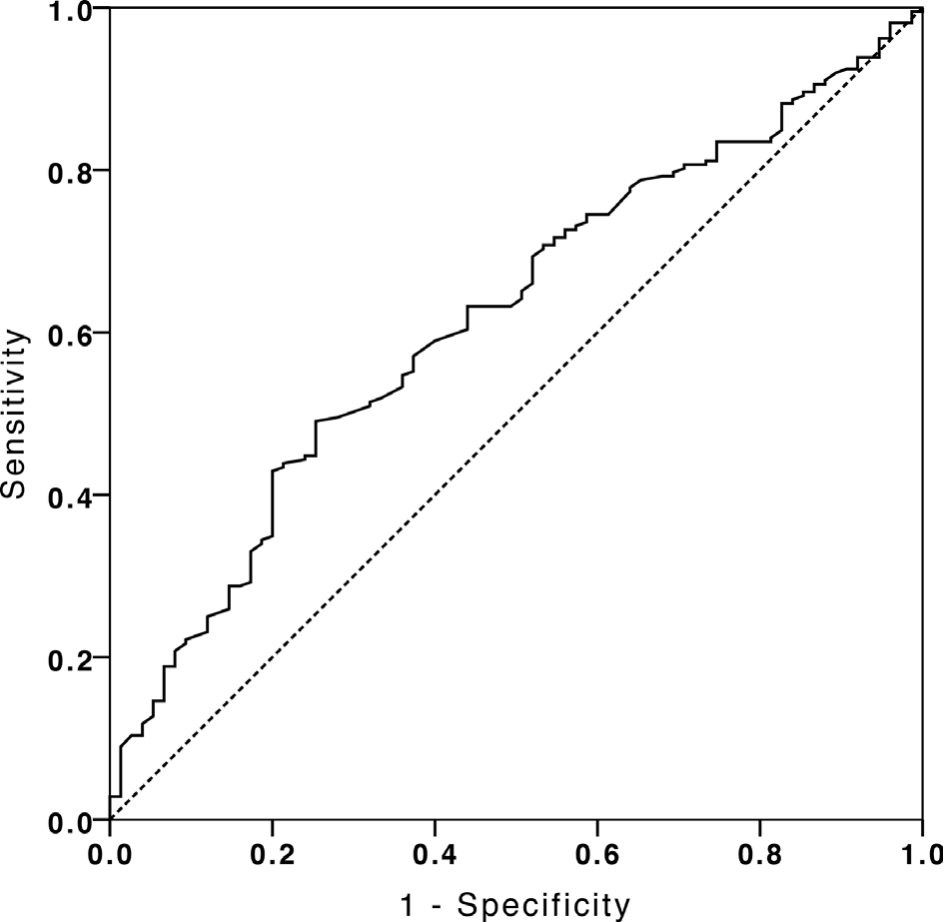
The receiver operating characteristic curve analysis of albumin for survival analysis.

The clinical characteristics of the 278 patients with DLBCL in the training cohort and 296 patients in the validation cohort were presented in **Table S1**. In our training cohort 260 (93.53%) patients achieved complete remission (CR), and 275 (92.91%) patients were CR in the validation cohort at EoT. There were no significant differences between the training cohort and validation cohort in the clinical features including gender, age, B symptoms, performance status, extranodal sites, stage, lactate dehydrogenase, cell of origin, IPI score, and albumin level at diagnosis (p > 0.05). According to ROC, the best cutoff value of albumin for survival analysis was 39.2 g/L with an area under the curve value of 0.618 ± 0.026 (p < 0.001, **Figure 1**). Low serum albumin was defined when albumin was less than 39.2 g/L. There were 356 patients (62.0%) with low serum albumin at the time of diagnosis, and these patients tended to present with older age (p = 0.021), poor performance status (p = 0.039), B symptoms (p < 0.011), high LDH (p < 0.001), advanced stage (p = 0.029), more extranodal sites (p = 0.034), and high IPI scores (p < 0.001). Baseline characteristics of these patients are displayed in the **Table 1**.

**Table 1:** Clinical characteristics of patients according to albumin in patients with DLBCL.

Characteristics	Total	Albumin		*P*-value
		Low	High	
Gender				0.649
Female	228(32.7%)	144(40.4%)	84(38.5%)	
Male	346(60.3%)	212(59.6%)	134(61.5%)	
Age				0.021
≤60y	456(79.4%)	272(76.4%)	184(84.4%)	
>60y	118(20.6%)	84(23.6%)	34(15.6%)	
Performance status				0.039
0–1	442(77.0%)	264(74.2%)	178(81.7%)	
2–4	132(23.0%)	92(25.8%)	40(18.3%)	
B symptoms				<0.001
No	412(71.8%)	226(63.5%)	186(85.3%)	
Yes	162(28.2%)	130(36.5%)	32(14.7%)	
Extranodal sites				0.034
0–1	294(51.2%)	170(47.8%)	124(56.9%)	
≥2	280(48.8%)	186(52.2%)	94(43.1%)	
Ann Arbor stage				0.029
I/II	210(36.3%)	118(33.1%)	92(42.2%)	
III/IV	364(63.4%)	238(66.9%)	126(57.8%)	
Lactate dehydrogenase				<0.001
Normal	102(17.8%)	46(12.9%)	56(25.7%)	
Elevated	472(82.2%)	310(87.1%)	162(74.3%)	
International prognostic index				<0.001
0–1	222(38.8%)	120(33.7%)	102(46.8%)	
2	132(23.0%)	80(22.5%)	52(23.9%)	
3	138(24.0%)	94(26.4%)	44(20.2%)	
4–5	82(14.3%)	62(17.4%)	20(9.2%)	
Albumin				<0.001
Low	228(39.7%)	172(48.3%)	56(25.7%)	
High	346(60.3%)	184(51.7%)	162(74.3%)	
Cell of origin				0.146
Germinal center B cell like	168(32.6%)	96(30.2%)	74(36.4%)	
Non-germinal center B cell like	348(67.4%)	222(69.8%)	126(63.6%)	

### Hypoalbuminemia After EoT Predicts Worse Prognosis

**Figure 2:**
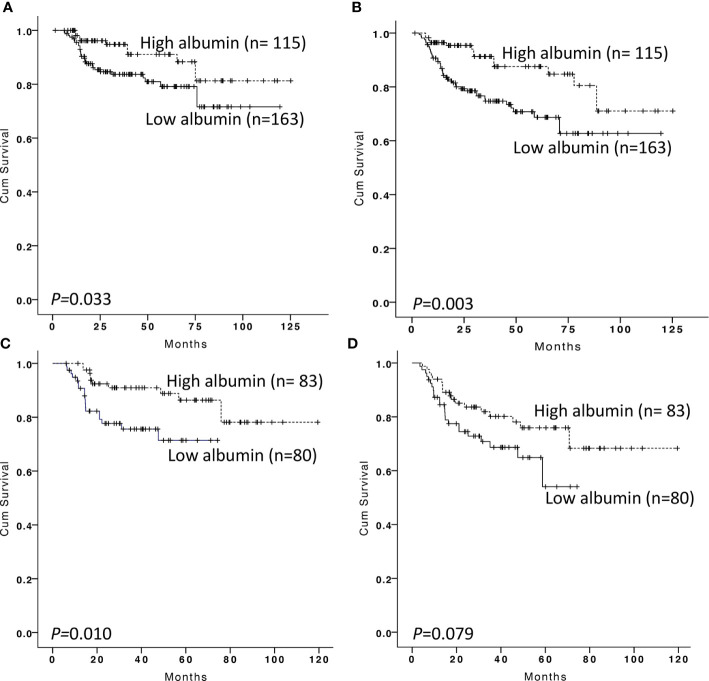
Kaplan–Meier survival analysis of overall survival and progression-free survival according to albumin at diagnosis and end-of treatment in the training cohort. Overall survival **(A)** and progression-free survival **(B)** according to albumin at diagnosis in DLBCL patients. Overall survival **(C)** and progression-free survival **(D)** according to albumin at the end of treatment in DLBCL patients with low serum albumin at diagnosis.

Two hundred and seventy-eight DLBCL patients were analyzed in our training cohort, and 163 of them were present with low serum albumin at the time of diagnosis. Survival analysis showed patients with low albumin showed more inferior OS and PFS than those with high albumin (p = 0.033, with 5-year OS of 79.1 ± 3.9 *versus* 91.1 ± 3.4%; p < 0.001, with 5-year PFS of 68.7 ± 4.5 *versus* 87.6 ± 3.9%, respectively, **Figures 2A, B**). To further investigate the clinical value of albumin changes after EoT, we explored the prognostic value of albumin change between diagnosis and EoT. The serum albumin was recovered in 83 of 163 patients after EoT, and the remaining 80 patients still had low serum albumin. The patient’s characteristics of those with serum albumin recovery *vs* those without were displayed in the **Table S2**. Our data showed patients with low albumin after EoT portended a worse OS and PFS than those with the recovery of albumin (p = 0.010, with 5-year OS of 71.4 ± 36.4 *versus* 86.4 ± 4.4%; p = 0.079, with 5-year PFS of 54.0 ± 11.3 *versus* 75.9 ± 5.3%, respectively, **Figures 2C, D**). Patients with albumin recovery after EoT showed no significant difference in OS and PFS compared with patients with high albumin at diagnosis (p = 0.569 and p = 0.075, respectively). The multivariable analysis revealed that low albumin after EoT was an unfavorable factor for OS [relative risk (RR), 1.959; 95% confidence interval (CI), 1.020–3.763, p = 0.043) and PFS (RR, 1.731; 95% CI, 1.014–2.957, p = 0.044) independent of IPI.

### Validation of Prognostic Value for Albumin Change After EoT

**Figure 3:**
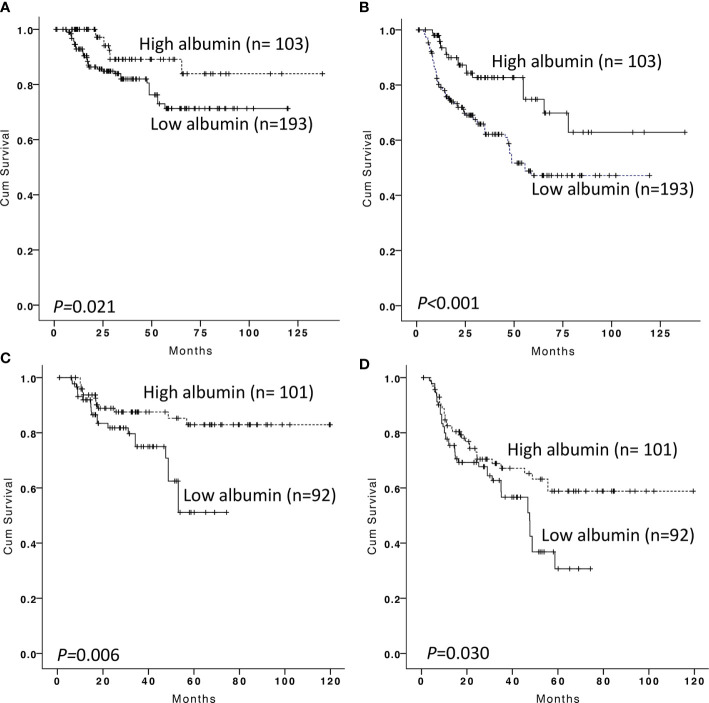
Kaplan–Meier survival analysis of overall survival and progression-free survival according to albumin at diagnosis and end-of treatment in the validation cohort. Overall survival **(A)** and progression-free survival **(B)** according to albumin at diagnosis in DLBCL patients. Overall survival **(C)** and progression-free survival **(D)** according to albumin at end of treatment in DLBCL patients with low serum albumin at diagnosis.

To validate the generalizability of the prognostic value of albumin changes in our model, 296 patients from the Second Affiliated Hospital of Shantou University Medical College and Changsha Central Hospital were used for external validation. There were 193 patients (65.2%) with low serum albumin at the time of diagnosis. Patients with low albumin at the time of diagnosis showed worse OS and PFS than those with high albumin (p = 0.021, with 5-year OS of 71.3 ± 34.7 *versus* 89.1 ± 3.9%; p < 0.001, with 5-year PFS of 47.2 ± 4.9 *versus* 74.8 ± 6.6%, respectively, **Figures 3A, B**). After EoT, serum albumin was recovered in 101 patients and the other 92 patients remained at the low serum albumin. We confirmed that 5-year OS and PFS in patients with low albumin after EoT were significantly lower than in those with albumin recovery (p = 0.006, with 5-year OS of 51.1 ± 9.8 *versus* 82.9 ± 4.6%; p = 0.030, with 5-year PFS of 30.7 ± 8.2 *versus* 58.8 ± 6.0%, respectively, **Figures 3C, D**). We also confirmed that low albumin after EoT was an unfavorable factor for OS (RR, 2.529; 95% CI, 1.372–4.659, p = 0.003) and PFS (RR, 2.153; 95% CI, 1.422–3.259, p < 0.001) independent of IPI by multivariable analysis. The multivariable survival analysis was shown in **Table 2**.

**Table 2:** Multivariable analysis of prognostic factors for survival.

Variable	OS			PFS		
	RR	95%CI	*P* value	RR	95%CI	*P* value
Covariables in the training cohort						
IPI	1.196	0.907–1.578	0.205	1.119	0.886–1.414	0.346
Albumin	1.959	1.020–3.763	0.043	1.731	1.014–2.957	0.044
Covariables in the validation cohort						
IPI	1.192	0.923–1.539	0.177	1.069	0.895–1.277	0.459
Albumin	2.529	1.372–4.659	0.003	2.153	1.422–3.259	<0.001
Covariables in the entire cohort						
IPI	1.195	0.990–1.443	0.064	1.092	0.948–1.259	0.222
Albumin	2.249	1.441–3.509	<0.001	2.001	1.443–2.773	<0.001

## Discussion

Low serum albumin at diagnosis has been identified as a simple prognostic factor in DLBCL before and after rituximab era (20, 21). One study with small sample also showed serum albumin was a stable biomarker over the course of treatment in DLBCL (18). But the prognostic value of albumin changes between diagnosis and EoT remained unknown. In this study, we retrospectively explored the prognostic value of albumin changes after R-CHOP regimen treatment in DLBCL patients and confirmed our results in another validation cohort. Our data showed low serum albumin at diagnosis and after EoT was associated with poor outcome, and albumin recovery after treatment implied superior survival.

The cutoff value of serum albumin level in our study was 39.2 g/L defined by ROC, which falls into the normal range of our hospital. According to serum albumin level, 58.6% of patients had a low serum albumin, and almost half of them still remained at the low serum albumin at EoT. Those patients showed poor OS and PFS compared with those with high serum albumin at diagnosis and EoT. We also explored the prognostic value of delta albumin between the time of diagnosis and EoT. Our data showed when albumin increased by more than 4.5 g/L after EoT, the patients with low albumin at diagnosis showed superior survival in both the training and validation cohorts (p = 0.001 and 0.001), but not in the patients with high albumin at diagnosis (p = 0.665 and 0.400) (data not shown). The underlying mechanisms of albumin related to the outcome of DLBCL remain unclear (22). Serum albumin level may be a surrogate of poor nutritional and inflammatory status as well as an aggressive tumor behavior (23–25). In our study, hypoalbuminemia was associated with more extranodal sites, advanced stage, high lactate dehydrogenase and also poor performance status and B symptoms, but not with the number of chemotherapy cycles. This indicated that hypoalbuminemia may be driven by the aggressive tumor behavior and inflammatory status rather than by poor nutritional status. Patients with albumin recovery at EoT may imply good disease control and hence outcome.

The major limitation of this study is its retrospective nature (22), which may introduce inherent selection bias by recruiting DLBCL patients who were diagnosed and followed at tertiary medical centers. To minimize the inherent biases of the retrospective study and ensure the homogeneity of treatment, we included only patients with *de novo* DLBCL treated with R-CHOP and excluded patients with primary central nervous system and mediastinal lymphoma, immunodeficiency-associated tumors, transformed non-Hodgkin lymphoma and posttransplant lymphoproliferative disorder, which might result in a selection bias. Despite these limitations, our results were validated in the validation cohort from different centers, which may improve reliability of our findings. As opposed to dynamic monitoring circulating tumor cells or DNA (26), serum albumin level has the advantages of ease of use, ready availability, and low cost.

In summary, we explored the prognostic value of albumin changes in patients with DLBCL and found low serum albumin at diagnosis and EoT was associated with poor outcome. Consecutive hypoalbuminemia is a simple and effective prognostic factor in DLBCL patients. Although the hypothesis requires more evidence to support, this reminds us to pay more attention to patients with low serum albumin at EoT during follow up.

## Data Availability Statement

The raw data supporting the conclusions of this article will be made available by the authors, without undue reservation.

## Ethics Statement

The studies involving human participants were reviewed and approved by Southern Medical University affiliated Nanfang Hospital before study initiation. The patients/participants provided their written informed consent to participate in this study.

## Author Contributions

RF and XW designed the study, analyzed and interpreted the data. JZ, ZZ, QL, MZ, WH, JC, QW, and YW collected data. RF and XW analyzed data and wrote the manuscript. All authors contributed to the article and approved the submitted version.

## Funding

This work was supported by the Outstanding Youth Development Scheme of Nanfang Hospital, Southern Medical University (Grant No. 2019J011), National Natural Science Foundation of Guangdong Province and Guangzhou City (Grant No. 2018A030313083 and 201804010351).

## Conflict of Interest

The authors declare that the research was conducted in the absence of any commercial or financial relationships that could be construed as a potential conflict of interest.
