# Supplementary material for: Consecutive Hypoalbuminemia Predicts Inferior Outcome in Patients With Diffuse Large B-Cell Lymphoma
Source: Front Oncol. 2021 Jan 27;10:610681. doi: 10.3389/fonc.2020.610681 (PMC7873605; doi:10.3389/fonc.2020.610681)
Supplement: Supplementary file 1 [file Table_1.docx]

Table S1. Clinical characteristics of patients according to training and validation group.

| Characteristics | Total | Training | | Validation | *P*-value |
| --- | --- | --- | --- | --- | --- |
| Gender |  |  |  | | 0.661 |
| Female | 346(60.3%) | 113(40.6%) | 115(38.9%) | |  |
| Male | 228(39.7%) | 165(59.4%) | 181(61.1%) | |  |
| Age |  |  |  | | 0204 |
| ≤60y | 456(79.4%) | 227(81.7%) | 229(77.4%) | |  |
| >60y | 118(20.6%) | 51(18.3%) | 67(22.6%) | |  |
| Performance status |  |  |  | | 0.832 |
| 0-1 | 442(77.0%) | 213(76.6%) | 229(77.4%) | |  |
| 2-4 | 132(23.0%) | 65(23.4%) | 67(22.6%) | |  |
| B symptoms |  |  |  | | 0.117 |
| No | 412(71.8%) | 208(74.8%) | 204(68.9%) | |  |
| Yes | 162(28.2%) | 70(25.2%) | 92(31.1%) | |  |
| Extranodal sites |  |  |  | | 0.442 |
| 0-1 | 294(51.2%) | 147(52.9%) | 147(49.7%) | |  |
| ≥2 | 280(48.8%) | 131(47.1%) | 149(50.3%) | |  |
| Ann Arbor stage |  |  |  | | 0.033 |
| Ⅰ/Ⅱ | 210(36.6%) | 114(41.0%) | 96(32.4%) | |  |
| Ⅲ/ Ⅳ | 364(63.4%) | 164(59.0%) | 200(67.6%) | |  |
| Lactate dehydrogenase |  |  |  | | 0.097 |
| Normal | 102(17.8%) | 57(20.5%) | 45(15.2%) | |  |
| Elevated | 472(82.2%) | 221(79.5%) | 251(84.8%) | |  |
| International prognostic index |  |  |  | | 0.104 |
| 0-1 | 222(38.7%) | 115(41.4%) | 107(36.1%) | |  |
| 2 | 132(23.0%) | 64(23.0%) | 68(23.0%) | |  |
| 3 | 138(24.0%) | 66(23.7%) | 72(24.3%) | |  |
| 4-5 | 82(14.3%) | 33(11.9%) | 49(16.6%) | |  |
| Albumin |  |  |  | | 0.105 |
| Low | 356(62.0%) | 163(58.6%) | 193(65.2%) | |  |
| High | 218(38.0%) | 115(41.4%) | 103(34.8%) | |  |
| Cell of origin |  |  |  | | 0.350 |
| Germinal center B cell like | 168(32.6%) | 88(34.5%) | 80(30.7%) | |  |
| Non-germinal center B cell like | 348(67.4%) | 167(65.5%) | 181(69.3%) | |  |
